# Point-of-Care Ultrasound to Detect Dilated Coronary Sinus in Adults

**DOI:** 10.24908/pocus.v7i2.15702

**Published:** 2022-11-21

**Authors:** Zouheir I Bitar, Mohamad Abdelfatah, Ossama Sajeh Maadarani, Muath Alanbaei, Rashed Juma Al Hamdan

**Affiliations:** 1 Consultant critical care medicine, Internal medicine department, Ahmadi hospital Kuwait; 2 Consultant critical care medicine, Internal medicine department, Ahmadi hospital Kuwait; 3 Critical care Unit, Ahmadi hospital, Kuwait oil company Kuwait; 4 Assistant Professor, faculty of medicine, Kuwait university Kuwait; 5 Team leader internal medicine and cardiologist, Ahmadi hospital, Kuwait oil company Kuwait

**Keywords:** echocardiography, arrhythmia

## Abstract

Detecting dilated coronary sinus when assessing patients in an acute emergency with point-of-care ultrasound (POCUS) is important for differential diagnosis, including the detection of persistent left superior vena cava (PLSVC) and right ventricular dysfunction. Cardiac POCUS with agitated saline injections through the left and right antecubital veins is a simple bedside test to make the diagnosis. We present a 42-year-old woman with first-time rapid atrial flutter in whom POCUS confirmed the presence of dilated coronary sinus and PLSVC.

## Introduction

Point-of-care ultrasound (POCUS), including cardiac examination, is widely used to assess patients with various cardiac and pulmonary emergencies, including chest pain, hypoxemia, shock, and arrhythmias 1. In critical care areas, POCUS is a fundamental examination to expedite the diagnostic evaluation in critically ill patients at the bedside and initiate urgent management by the physician 2. An unexpected emergency POCUS finding can direct us to the correct diagnosis and impact management. This case report presents a patient with first-time atrial fibrillation (AF) in whom cardiac POCUS revealed a dilated coronary sinus. Adding bedside testing with agitated saline contrast assists with the overall diagnosis of complicated adult congenital heart disease.

## Case report

A 42-year-old married woman G7 P7, not known to be diabetic or hypertensive and not on any medications, presented to the emergency room with first-time tachycardia for 2 hours. Her blood pressure was 120/70  mHg, her heart rate was 120 beats/minute, and it was irregular. There was right ventricular heaving and soft systolic murmur in three of six in the left parasternal line of the intercostal space on chest examination. An electrocardiographic study revealed a right axis with rapid AF.

**Figure 1:**
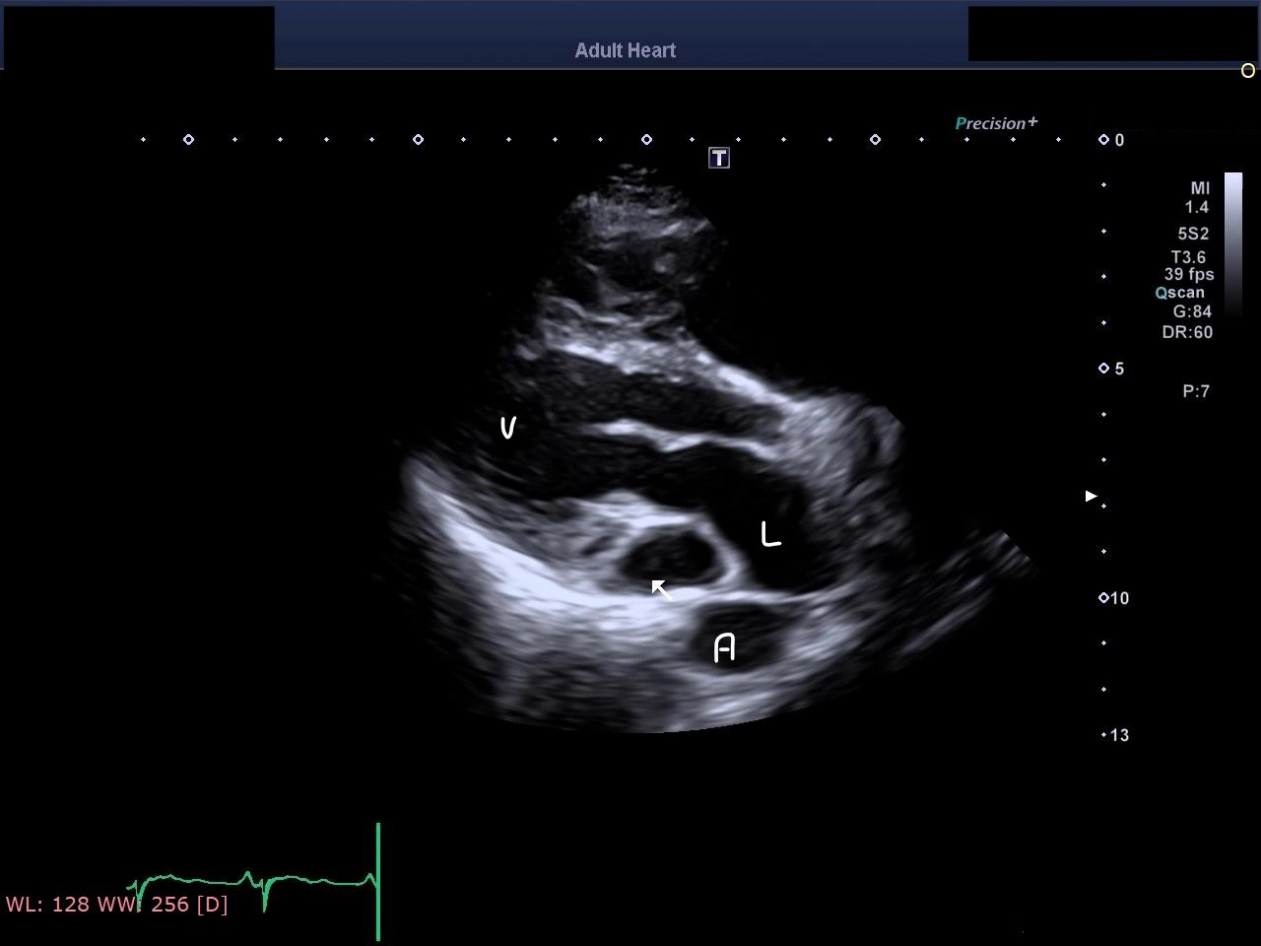
Dilated coronary sinus. Arrow, coronary sinus; L, left atrium; V, left ventricle

In an attempt to control the heart rate with verapamil, 5 mg was intravenously administered, and she spontaneously reverted to sinus rhythm after 4 hours. POCUS revealed dilatation of the coronary sinus to a diameter of 16 mm (Figure 1 and online Video S1). A phased-array 5-MHz ultrasound probe (GE Vivid S6N, N-3191 Horten, Norway) was used. Dilatation of the coronary sinus raised our suspicion of persistent left superior vena cava (PLSVC). In addition, POCUS revealed no echo drop-out in the interatrial, septum, normal size, or systolic function of the left ventricle (LV) or dilated right ventricle (RV).

**Figure 2:**
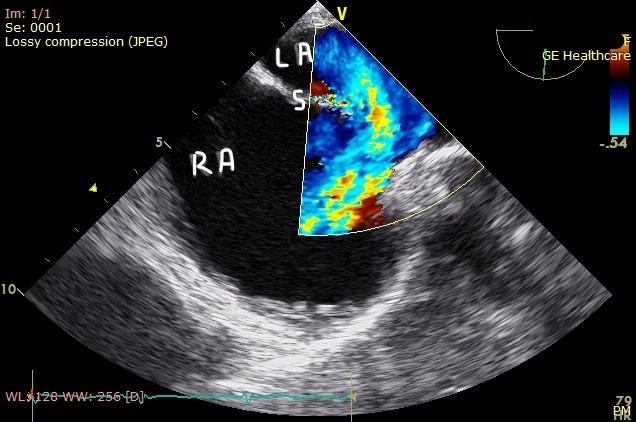
Transesophageal echocardiography showed an atrial septal defect of the superior sinus venosus type with Left to Right flow. RA, right atrium; LA left atrium; S atrium septum with septum defect

An agitated saline contrast POCUS was then performed. Cardiac POCUS during injection of saline bubbles from the left cubital vein showed bubbles entering the coronary sinus and then the right atrium (RA; online Video S2). When injection of the saline bubble was performed from the right cubital vein, the agitated saline contrast enhanced the RA before the coronary sinus, suggesting PLSVC with normal right superior vena cava (SVC). Transesophageal echocardiography showed an atrial septal defect of the superior sinus venosus type with left-to-right flow (Figure 2). The RA and the RV were dilated. She had moderate tricuspid regurgitation with an estimated pulmonary artery pressure of 40 mmHg. After agitation with saline, the bubbles failed to cross the left atrium (LA) through that presumed defect. The left and right lower pulmonary veins could be seen, but the upper ones could not. The RV and LV ejection fractions were preserved.

**Figure 3:**
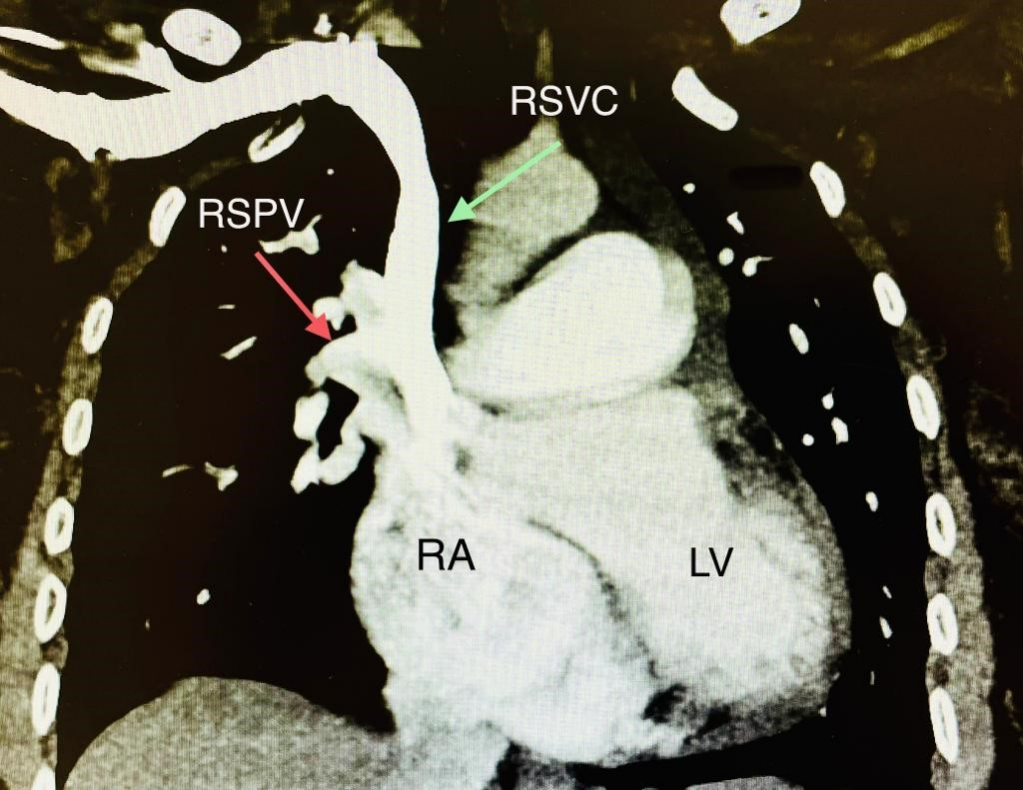
Cardiac computerized tomography with emphasis on pulmonary veins was performed. The right atrium (RA) and right ventricle are significantly dilated. There is an anomalous shunt defect at the superior portion of the interatrial Septum. The right superior pulmonary vein (RSPV) is seen having confluence with the right superior vena cava (RSVC) at the level of entrance of RSPV into the mediastinum.

Cardiac computerized tomography with an emphasis on pulmonary veins was performed (Figure 3). The RA and RV were significantly dilated, with a prominent RA appendage and multiple trabeculations in the RV with stretching/thinning of the wall. There was an anomalous shunt defect at the superior portion of the interatrial septum. The right superior pulmonary vein (RSPV) was seen to have confluence with the right SVC at the level of entrance of the RSPV into the mediastinum. The combined common confluence/channel of the RSPV and SVC were together seen entering and communicating into the RA and LA, thereby forming an anomalous shunt defect. The persistent left SVC drained into the prominent coronary sinus (16 mm) and, in turn, drained into the RA (normal anatomical communication). This dilated pulmonary arterial system up to the involvement of the segmental pulmonary artery branches resulted in pulmonary arterial hypertension.

Cardiac catheterization was performed under local anesthesia. Access was achieved via the right femoral vein and artery approach. The following saturations were measured (all in air): high SVC 62.6%, low SVC 65.6%, RA 85.5%, pulmonary artery 86.6%, RV 87.4%, and right femoral artery 95.5%. The pressures were the following: RA mean pressure 3-5 mmHg, pulmonary artery mean pressure 14 mmHg, pulmonary artery wedge pressure 5 mmHg, and LV end-diastolic pressure 8 mmHg. The pressure from the LV to the ascending aorta to the descending aorta showed no gradient. The selective coronary angiogram was normal. These data indicated a large right-to-left shunt with normal coronary arteries. 

The results indicated large left-to-right shunt normal coronaries with low pulmonary artery pressure. The diagnosis was confirmed as superior sinus venosus atrial septal defect (ASD) with a bilateral SVC. The situation was complicated by right heart enlargement resulting in severe tricuspid regurgitation. The surgical repair included the closure of the superior sinus venosus atrial septal defect, rerouting pulmonary venous blood toward the LA, and repairing the tricuspid valve.

The operation was uneventful; follow-up showed no more AF, and follow-up echocardiography 6 months later showed normal RA and RV.

## Discussion

Five to 10 percent of ASDs are sinus venosus defects and lie in the venous part of the atrial septum 3. Sinus venosus defects, whether the superior or inferior type, are abnormal insertions of the superior or inferior vena cava that override the interatrial septum. Interatrial communication is then formed within the insertion of the overriding vein 4. Thus, sinus venosus defects are technically not ASDs since the defect is within the sinus venosus septum. An anomalous connection involving one or more pulmonary veins is present in most patients with sinus venosus ASD 5.

The common complications of ASDs include atrial arrhythmias, pulmonary hypertension, and paradoxical embolism. Atrial arrhythmias, particularly AF, often come along with ASDs after the third decade of life. Atrial fibrillation or atrial flutter is present in almost 20% of patients with ASDs, increasing with age and with pulmonary hypertension 6. In a report of 211 adults, the incidence of atrial fibrillation or atrial flutter prior to surgery was 1% among those aged 18 to 40, 30% among those aged 40 to 60, and 80% among those over the age of 60 years 6. Our patient was 42 years old at her first presentation. The patient tolerated seven pregnancies, with her congenital heart disease undetected till we used POCUS in acute AF. Women with cardiovascular disease are undertreated, and POCUS may have an important role to play in bridging this disparity of care 7.

Point-of-care cardiac ultrasound assists in answering a specific clinical question and narrowing the differential diagnosis in critically ill patients at the bedside in a time-sensitive manner. The managing physician usually performs and interprets the cardiac ultrasound findings. By contrast, comprehensive echocardiography is performed by a certified echocardiographer in an echocardiography laboratory and interpreted by a cardiologist 8. The patient in the current case report presented for the first time with rapid AF and POCUS, identified an important structural, and congenital heart disease.

POCUS is an important tool for detecting dilated coronary sinus abnormalities in acute emergencies. A right coronary sinus is rarely seen in healthy individuals unless it is dilated and located in the left posterior atrioventricular groove 4. Coronary sinus dilatation is a sign of impaired right ventricular function in patients with heart failure 9. However, a dilated right coronary sinus may be visualized in patients with congestive heart failure, pulmonary hypertension, RV failure, PLSVC, or total anomalous pulmonary venous return 5. However, in the acute setting, the intensivist should be aware of its association with a PLSVC, as this has been associated with difficulties in central venous line placement and adult congenital heart disease with intracardiac shunts 10. Therefore, it is prudent to conduct agitated saline contrast while doing cardiac POCUS at the bedside to confirm the diagnosis of PLSVC. A PLSVC drains directly into the coronary sinus, leading to a characteristic sequence of contrast appearance. Following injection of contrast into a left arm vein, the contrast appears in the coronary sinus before appearing in the RA 9. Upon intravenous injection of contrast into the right arm, there is normal transit of contrast with right atrial opacification before the contrast is seen in the coronary sinus.

## Conclusion

This case illustrates the importance of recognizing the causes of dilated coronary sinus detected by POCUS and completing the exam with agitated saline contrast. Congenital heart disease, such as sinus venosus defect, can present in adulthood and should be considered in the differential diagnosis when evaluating patients in critical care areas by POCUS. The patient tolerated seven pregnancies, with her congenital heart disease undetected till we used POCUS in acute AF. 

## Conflict of interest

The authors declare no conflicts of interest.

## Sources of funding

No funding was obtained for this study.

## Consent

Written informed consent was obtained from the patient for publication of this case report and accompanying images. 

## Supplementary Material

 Video S1 Showing dilated coronary sinus. Arrow, coronary sinus; LA , left atrium; LV left ventricle; Ao descending aorta.

Video S2 Cardiac POCUS imaging during injection of saline bubble from the left cubital vein showed bubbles entering the coronary sinus and then the right atrium and ventricle. arrow : coronary sinus with saline air bubles; R, Right ventricles with saline air bubbles ; A, aortic valve; Ao, descending aorta.

## References

[R165878426892824] Perera P, Mailhot T, Riley D, Mandavia D (2010). The RUSH exam: Rapid Ultrasound in SHock in the evaluation of the critically lll. Emerg Med Clin North Am.

[R165878426892833] Labovitz A J, Noble V E, Bierig M, Goldstein S A, Jones R, Kort S, Porter T R, Spencer K T, Tayal V S, Wei K (2010). Focused cardiac ultrasound in the emergent setting: a consensus statement of the American Society of Echocardiography and American College of Emergency Physicians. J AmSoc Echocardiogr.

[R165878426892825] Zaghal A M al, Li J, Anderson R H, Lincoln C, Shore D, Rigby M L (1997). Anatomical criteria for the diagnosis of sinus venosus defects. Heart.

[R165878426892832] Berger F, Vogel M, Kramer A, Weng Y, Lange P E, Hetzer R (1999). Incidence of atrial flutter/fibrillation in adults with atrial septal defect before and after surgery. Ann Thorac Surg.

[R165878426892826] Jost C H Attenhofer, Connolly H M, Danielson G K, Bailey K R, Schaff H V, Shen W K, Warnes C A, Seward J B, Puga F J, Tajik A J (2005). Sinus venosus atrial septal defect: long-term postoperative outcome for 115 patients. Circulation.

[R165878426892828] Çakıcı M, Doğan A, Çetin M, Süner A, Polat M, Oylumlu M, Aktürk E, Abus S, Üçkardeş F (2015). Coronary sinus dilatation is a sign of impaired right ventricular function in patients with heart failure. Anatol J Cardiol.

[R165878426892829] Wenger N K, Lloyd-Jones D M, Elkind Msv, Fonarow G C, Warner J J, Alger H M, Cheng S, Kinzy C, Hall J L, Roger V L, Association American Heart (2022). Call to Action for Cardiovascular Disease in Women: Epidemiology, Awareness, Access, and Delivery of Equitable Health Care: A Presidential Advisory from the American Heart Association. Circulation.

[R165878426892831] Weekes A J, Quirke D P (2011). Emergency echocardiography. Emerg Med Clin North Am.

[R165878426892827] Stewart J A, Fraker T D, Slosky D A, Wise N K, Kisslo J A (1979). Detection of persistent left superior vena cava by two-dimensional contrast echocardiography. J Clin Ultrasound.

[R165878426892830] Fares W H, Birchard K R, Yankaskas J R (2011). Persistent left superior vena cava identified during central line placement: a case report. Respir Med CME.

